# Endothelial Nitric Oxide Synthase Gene G894T Polymorphism and Myocardial Infarction: A Meta-Analysis of 34 Studies Involving 21068 Subjects

**DOI:** 10.1371/journal.pone.0087196

**Published:** 2014-01-30

**Authors:** Jian-Quan Luo, Jia-Gen Wen, Hong-Hao Zhou, Xiao-Ping Chen, Wei Zhang

**Affiliations:** Pharmacogenetics Research Institute, Institute of Clinical Pharmacology, Hunan Key Laboratory of Pharmacogenetics, Central South University, Changsha, P.R. China; MOE Key Laboratory of Environment and Health, School of Public Health, Tongji Medical College, Huazhong University of Science and Technology, China

## Abstract

**Background:**

Researches have revealed that the endothelial nitric oxide synthase (*eNOS*) gene G894T polymorphism is associated with the risk of Myocardial infarction (MI), but the results remain conflicting.

**Objective and Methods:**

A meta-analysis was conducted to investigate the association between *eNOS* G894T polymorphism and MI. Published studies from PubMed, Embase, CNKI and CBM databases were retrieved. The pooled odds ratios (ORs) for the association between *eNOS* G894T polymorphism and MI and their corresponding 95% confidence intervals (CIs) were estimated using the random- or fixed- effect model.

**Results:**

A total of 34 studies including 8229 cases and 12839 controls were identified for the meta-analysis. The *eNOS* G894T polymorphism was significantly associated with MI under a homozygous genetic model (OR = 1.41, 95% CI = 1.08–1.84; *P* = 0.012), a recessive genetic model (OR = 1.35, 95% CI = 1.06–1.70; *P* = 0.014), a dominant genetic model (OR = 1.18, 95% CI = 1.04–1.34; *P* = 0.009). In the subgroup analysis by ethnicity (non-Asian and Asian), no significant association was observed between *eNOS* G894T polymorphism and MI risk among non-Asians (*P*>0.05), but a positive significant association was found among Asians (*P*<0.05).

**Conclusions:**

The *eNOS* G894T polymorphism is associated with increased MI risk in Asians. The results indicate that ethnicity plays important roles in the association between *eNOS* G894T polymorphism and MI.

## Introduction

Myocardial infarction (MI) is a complex syndrome determined by multiple predisposing genetic and environmental factors. Previous studies have investigated the association of genetic variants in DNA repair pathways, lipid-related pathways, fibrinolytic system, renin angiotensin aldosterone system and nitric oxide synthase with MI risk [1], [2], [3], [4], [5].

There are several forms of nitric oxide synthase such as neuronal nitric oxide synthase (nNOS), endothelial nitric oxide synthase (*eNOS*), inducible nitric oxide synthase (iNOS). The vascular nitric oxide (NO), mainly produced by eNOS, is a critical molecule in regulating the vascular system, including the inhibition of the platelet aggregation and adhesion and reduction of vascular smooth muscle cell proliferation [6]. Furthermore, overproduction of NO can inhibit DNA repair and cause DNA damage [7], which plays an important role in the occurrence of MI [3]. NO regulation may result from the functional eNOS genetic polymorphisms. The eNOS gene is mapped on human chromosome 7q35–36 and contains 26 exons and 25 introns. The *eNOS* G894T polymorphism, a coding region variant, results in a Glu298Asp substitution and decreases the NO levels [8].

To date, studies on the association of *eNOS* G894T polymorphism with MI clinical phenotype have been extensively explored. However, the results still remain inconclusive and conflicting. Some studies found that the allele T of *eNOS* G894T polymorphism was the risk factor for MI, but others had the opposite results. Therefore, in the current study, a meta-analysis from 34 individual studies with a total of 21068 subjects including 8229 cases and 12839 controls was performed to get a more precise estimation of the association between *eNOS* G894T polymorphism and MI.

## Materials and Methods

### Publication Search and Inclusion Criteria

We searched the electronic databases PubMed, Embase, Chinese National Knowledge Infrastructure (CNKI), and Chinese Biomedical Literature Database (CBM) using the following search terms: (myocardial infarction or myocardial infarct) and (endothelial nitric oxide synthase) and (polymorphism or mutation or variant), without restriction on language. The included articles were published before September 2013. All eligible studies were retrieved, and their references were examined manually for other potentially relevant studies.

The inclusion criteria were as follows: a) case-control design. b) the association of *eNOS* G894T polymorphism with MI should be evaluated. c) the genotype data was available in the cases and controls. d) the control subjects must be in agreement with the Hardy-Weinberg equilibrium (HWE).

### Data Extraction

All data were independently collected from the included studies according to a standardized protocol by two investigators. The discrepancies during data extraction were resolved by consensus. The same data in different studies were used only once. The following information was extracted: first author’s name, publication year, original country, ethnicity, sample size, and number of genotype in cases and controls.

### Statistical Analysis

The association between *eNOS* G894T polymorphism and MI was assessed using crude odds ratio (OR) with 95% confidence interval (CI). The pooled ORs were determined for homozygous model (TT versus GG), heterozygous model (GT versus GG), recessive model (TT versus GT/GG), dominant model (GT/TT versus GG). The Z test was used to assess the pooled OR with the significance set at P<0.05. HWE was assessed using the Chi-square test in control groups. The presence of between-study heterogeneity was evaluated by using the I^2^ statistic test, which does not inherently depend on the number of studies in the meta-analysis and is preferable to the test of heterogeneity [9]. The value of I^2^ ranged from 0–100%. If obvious heterogeneity was observed among the studies (I^2^>50%), the random-effects model (the DerSimonian and Laird method) was used to calculate the pooled OR and 95% CI [10]. Otherwise, the fixed-effects model (the Mantel-Haenszel method) was adopted for the meta-analysis [11]. Subgroup analyses according to the ethnicity and the total sample size were also performed to evaluate the association. When stratified by total sample size, we defined the large group if the sample size was more than 1000 and the small group if the sample size was less than 400, otherwise was the medium group. Meta-regression was performed to explore the sources of between-study heterogeneity. The study ethnicity, total sample size, control sample size, MI sample size, ratio of MI sample size to control sample size, publication year were regarded as the potential confounding factors. Sensitivity analyses were conducted to evaluate the effect of individual study on pooled results and assess the stability of results. The potential publication bias was detected with Begg’s funnel plot [12], and the funnel plot asymmetry was assessed by Egger’s linear regression test [13]. All statistical analyses were performed using the STATA 12.0 software (StataCorp, College Station, TX, USA).

## Results

### Characteristics of Eligible Studies

Our meta-analysis was performed according to guidelines of the “Preferred Reporting Items for Systematic reviews and Meta-Analyses” (PRISMA) statement (Supplement S1) [14]. A total of 417 relevant papers were yielded by the literature search, among which 34 studies met the inclusion criteria, including 8229 MI cases and 12839 controls. As is showed in the flow diagram (Supplement S2), 372 papers were excluded owing to the obvious irrelevance. We reviewed the full texts of the remaining 45 articles. Among them, 2 were reviews, 4 were duplicated publications, 3 had no controls, 4 had insufficient data for calculation of OR and 95% CI and 1 was deviated from the HWE. At last, a total of 34 studies for the association between *eNOS* G894T polymorphism and MI risk were obtained in the final meta-analysis. Data collected from the included studies were summarized in the Table 1. Those included studies in Japan, Australia, France, Northern Ireland, United Kingdom, Germany, Turkey, Greece, Korea, Brazil, Sweden, Poland, Hungary, Mexico, India, Egypt, Netherlands and China.

**Table 1:** Characteristics of eligible studies included in the meta-analysis.

First author	Year	Country	Ethnicity	Sample size (Case/Control)	MI			Control			MAF	HWE of control
					GG	GT	TT	GG	GT	TT		
Shimasaki et al [15]	1998	Japan	Asian	285/607	225	59	1	526	80	1	0.068	0.254
Hibi et al [16]	1998	Japan	Asian	226/357	189	32	5	295	62	0	0.087	0.072
Cai et al [17]	1998	Australia	Caucasian	95/478	54	35	6	244	197	37	0.283	0.751
Poirier et al [18]	1999	France	Caucasian	368/421	163	156	49	148	219	54	0.388	0.051
Poirier et al [18]	1999	Northern Ireland	Caucasian	163/155	55	76	32	58	72	25	0.394	0.738
Hingorani et al [19]	1999	United Kingdom	Caucasian	249/183	97	107	45	86	81	16	0.309	0.617
Cai et al [20]	1999	Australia	Caucasian	306/457	134	137	35	220	182	55	0.319	0.072
Song et al [21]	2000	China	Asian	114/104	89	18	7	90	13	1	0.072	0.501
Wang et al [22]	2001	Taiwan	Asian	114/218	97	17	0	177	38	3	0.101	0.560
Gardemann et al [23]	2002	Germany	Caucasian	1277/533	565	561	151	256	227	50	0.307	0.975
Wei et al [24]	2002	China	Asian	51/108	40	9	2	98	10	0	0.046	0.614
Aras et al [25]	2002	Turkey	Caucasian	76/117	43	28	5	60	48	9	0.282	0.888
Qi et al [26]	2003	China	Asian	107/81	82	16	9	68	13	0	0.080	0.432
Schmoelzer et al [27]	2003	Austria	Caucasian	126/248	60	54	12	121	102	25	0.306	0.609
Agema et al [28]	2004	Netherlands	Caucasian	356/574	174	157	25	216	270	88	0.389	0.811
Zhan et al [29]	2005	China	Asian	37/172	25	12	0	141	31	0	0.090	0.194
Antoniades et al [30]	2005	Greece	Caucasian	228/519	97	99	32	255	217	47	0.300	0.932
Yu et al [31]	2006	China	Asian	120/264	98	22	0	237	26	1	0.053	0.752
Chao et al [32]	2006	China	Asian	41/150	25	11	5	119	29	2	0.110	0.877
Jo et al [33]	2006	Korea	Asian	129/803	104	23	2	667	131	5	0.088	0.600
Sampaio et al [34]	2007	Brazil	Mixed	115/104	56	46	13	52	45	7	0.284	0.509
Andrikopoulos et al [35]	2008	Greece	Caucasian	1602/727	722	701	179	352	297	78	0.312	0.199
Odeberg et al [36]	2008	Sweden	Caucasian	318/85	179	121	18	43	32	10	0.306	0.296
Vasilakou et al [37]	2008	Greece	Caucasian	49/161	30	16	3	76	74	11	0.298	0.212
Gluba et al [38]	2009	Poland	Caucasian	278/134	140	118	20	62	61	11	0.311	0.454
Szabó et al [39]	2009	Hungary	Caucasian	118/384	39	58	21	200	161	23	0.270	0.204
Isordia-Salas et al [40]	2010	Mexico	Mixed	180/180	104	62	14	134	42	4	0.139	0.742
Angeline et al [41]	2010	India	Asian	100/100	56	30	14	67	31	2	0.175	0.462
Dafni et al [42]	2010	Greece	Caucasian	204/218	83	94	27	108	95	15	0.287	0.334
Katakami et al [43]	2010	Japan	Asian	226/3593	182	43	1	3045	533	15	0.078	0.103
Gad et al [44]	2012	Egypt	Caucasian	104/101	52	47	5	59	34	8	0.248	0.333
Zigra et al [45]	2013	Greece	Caucasian	107/103	50	46	11	50	42	11	0.312	0.626
Narne et al [46]	2013	India	Asian	73/121	42	29	2	84	35	2	0.162	0.442
Arun et al [47]	2013	India	Asian	287/279	213	62	12	190	82	7	0.172	0.597

MI: Myocardial infarction; MAF: minor allele frequency; HWE: Hardy-Weinberg equilibrium.

### Results of Meta-analysis

A significant association between *eNOS* G894T polymorphism and MI was found under a homozygous genetic model (OR = 1.41, 95% CI = 1.08–1.84; *P* = 0.012), a heterozygous genetic model (OR = 1.12, 95% CI = 1.00–1.25; *P* = 0.054), a recessive genetic model (OR = 1.35, 95% CI = 1.06–1.70; *P* = 0.014), a dominant genetic model (OR = 1.18, 95% CI = 1.04–1.34; *P* = 0.009) (Table 2).

**Table 2:** Pooled ORs and 95% CIs of the association between eNOS G894T polymorphism and MI.

	TT vs. GG			GT vs. GG			TT vs. GT/GG			TT/GT vs. GG		
	OR (95% CI)	I^2^ (%)	*P*-value	OR(95% CI)	I^2^ (%)	*P*-value	OR(95% CI)	I^2^ (%)	*P*-value	OR(95% CI)	I^2^ (%)	*P*-value
Overall	1.41(1.08–1.84)	67.1	0.012	1.12(1.00–1.25)	53.4	0.054	1.34(1.06–1.70)	60.9	0.014	1.18(1.04–1.34)	65.6	0.009
Ethnicity												
Asian	3.44(2.15–5.49)	19.3	0.000	1.26(1.02–1.57)	50.1	0.032	3.41(2.14–5.43)	14.1	0.000	1.40(1.13–1.74)	52.8	0.002
Non-Asian	1.18(0.89–1.55)	72.8	0.250	1.05(0.93–1.20)	54.4	0.430	1.15(0.91–1.46)	65.4	0.236	1.08(0.93–1.25)	69.1	0.322
Sample size												
Small	1.67(1.26–2.21)	48.0	0.000	1.24(1.07–1.43)	28.7	0.005	1.58(1.21–2.07)	45.1	0.001	1.32(1.15–1.52)	45.8	0.000
Medium	1.30(0.83–2.03)	80.1	0.256	1.01(0.84–1.21)	67.5	0.902	1.27(0.87–1.86)	75.0	0.221	1.05(0.86–1.29)	76.5	0.626
Large	1.22(0.97–1.52)	0.0	0.087	1.16(1.02–1.33)	0.0	0.023	1.14(0.92–1.42)	0.0	0.219	1.18(1.04–1.33)	0.0	0.011

OR: odds ratio; 95% CI: 95% confidence interval. P-value was for pooled ORs. When I^2^<50%, it was for fixed effect model, otherwise it was for random effect model. Small study: studies with less than 400 participants; Medium study: studies with more than 400 and less than 1000 participants; Large study: studies with more than 1000 participants.

**Figure 1:**
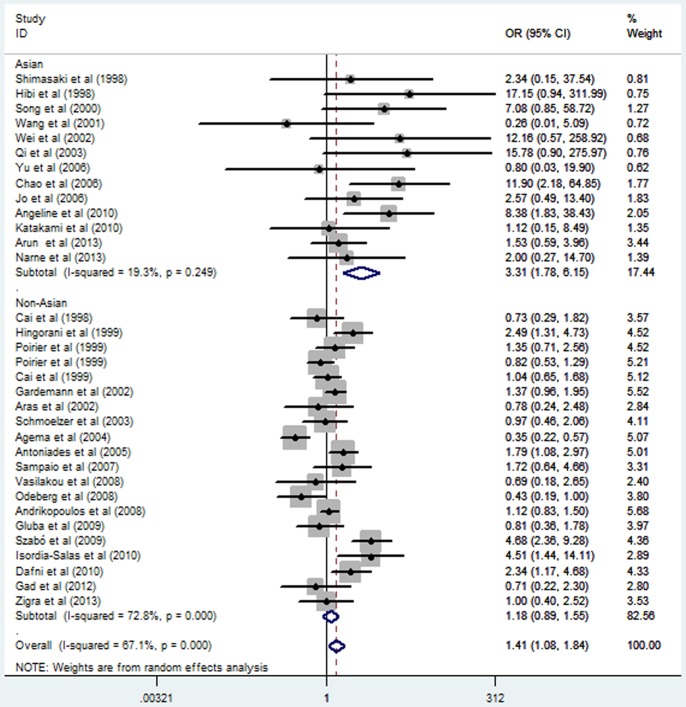
Forest plot of myocardial infarction associated with eNOS G894T polymorphism under a homozygous genetic model (TT vs. GG) stratified by ethnicity.

**Figure 2:**
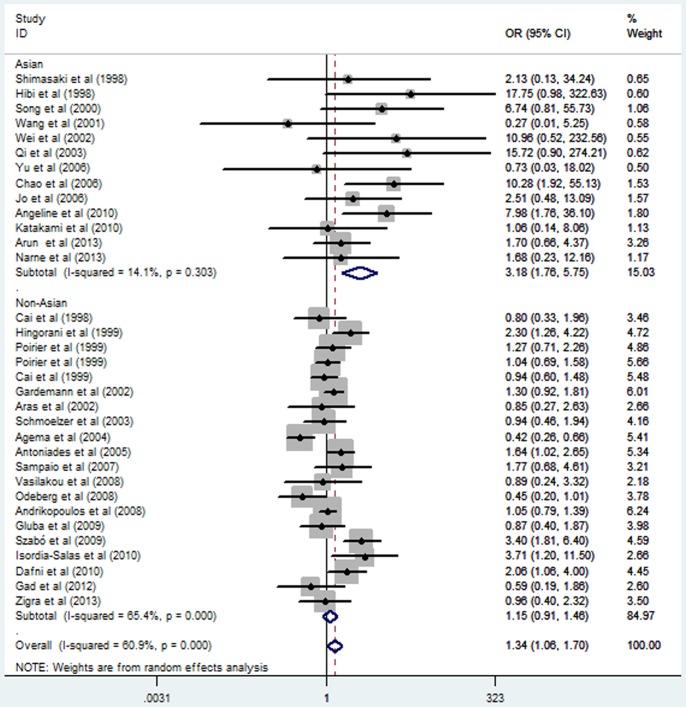
Forest plot of myocardial infarction associated with eNOS G894T polymorphism under a recessive genetic model (TT vs. GG/GT) stratified by ethnicity.

**Figure 3:**
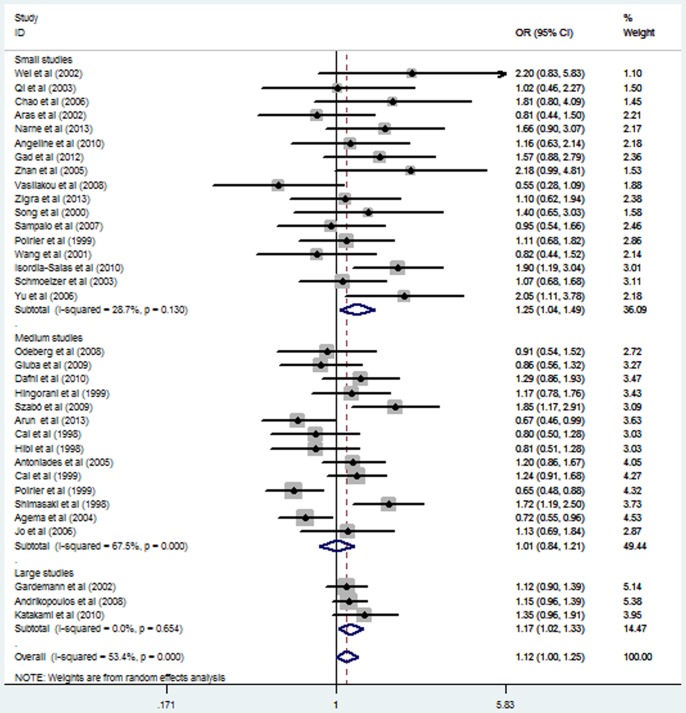
Forest plot of myocardial infarction associated with eNOS G894T polymorphism under a heterozygous genetic model (GT vs. GG) stratified by the total sample size.

**Figure 4:**
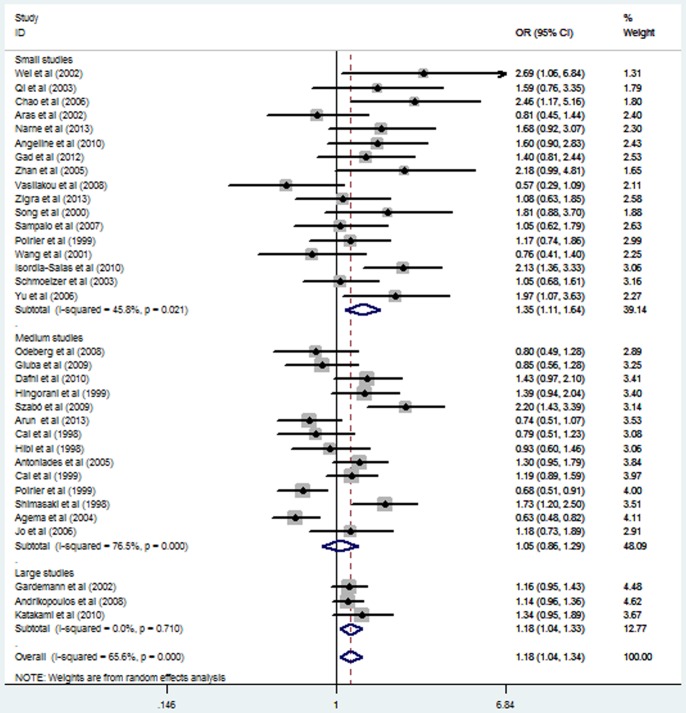
Forest plot of myocardial infarction associated with eNOS G894T polymorphism under a dominant genetic model (TT/GT vs. GG) stratified by the total sample size.

Subgroup analysis stratified by ethnicity also suggested a significant association between *eNOS* G894T polymorphism and MI in the Asian subgroup under a homozygous genetic model (OR = 3.44, 95% CI = 2.15–5.49; *P* = 0.000), a heterozygous genetic model (OR = 1.26, 95% CI = 1.02–1.57; *P* = 0.032), a recessive genetic model (OR = 3.41, 95% CI = 2.14–5.43; *P* = 0.000), and a dominant genetic model (OR = 1.40, 95% CI = 1.13–1.74; *P* = 0.002). In contrast, no significant association was observed in the non-Asian subgroup under any of the genetic models (*P>0.05*) (Table 2; Figures 1 and 2;Supplements S3 and S4). Stratified analyses by the total sample size also suggested that *eNOS* G894T polymorphism increased the MI risk both in large sample size studies and small sample size studies (Table 2; Figures 3 and 4).

### Sources of Heterogeneity

Under homozygous and recessive genetic models, meta-regression revealed that ethnicity was the sources of between-study heterogeneity (*P* = 0.007, *P* = 0.004 respectively), which was consistent with subgroup analyses results in homozygous and recessive genetic models (Table 2; Figures 1 and 2). Moreover, under the dominant genetic model, meta-regression showed that ethnicity might be the sources of between-study heterogeneity (*P* = 0.058), which was also consistent with subgroup analyses results in the dominant genetic model (Table 2). In addition, subgroup analyses revealed that the heterogeneity was significantly reduced in the small sample size group and large sample size group in all genetic models, suggesting that the total sample size was the source of heterogeneity (Table 2; Figures 3 and 4).

### Sensitivity Analysis

A single study was excluded each time to evaluate the effect of individual study on the combined ORs and 95% CIs. The omission of any single study did not make significant difference in the pooled effects of homozygous, heterozygous, recessive and dominant genetic models, suggesting a high stability of our meta-analysis results (data not shown).

### Publication Bias

**Figure 5:**
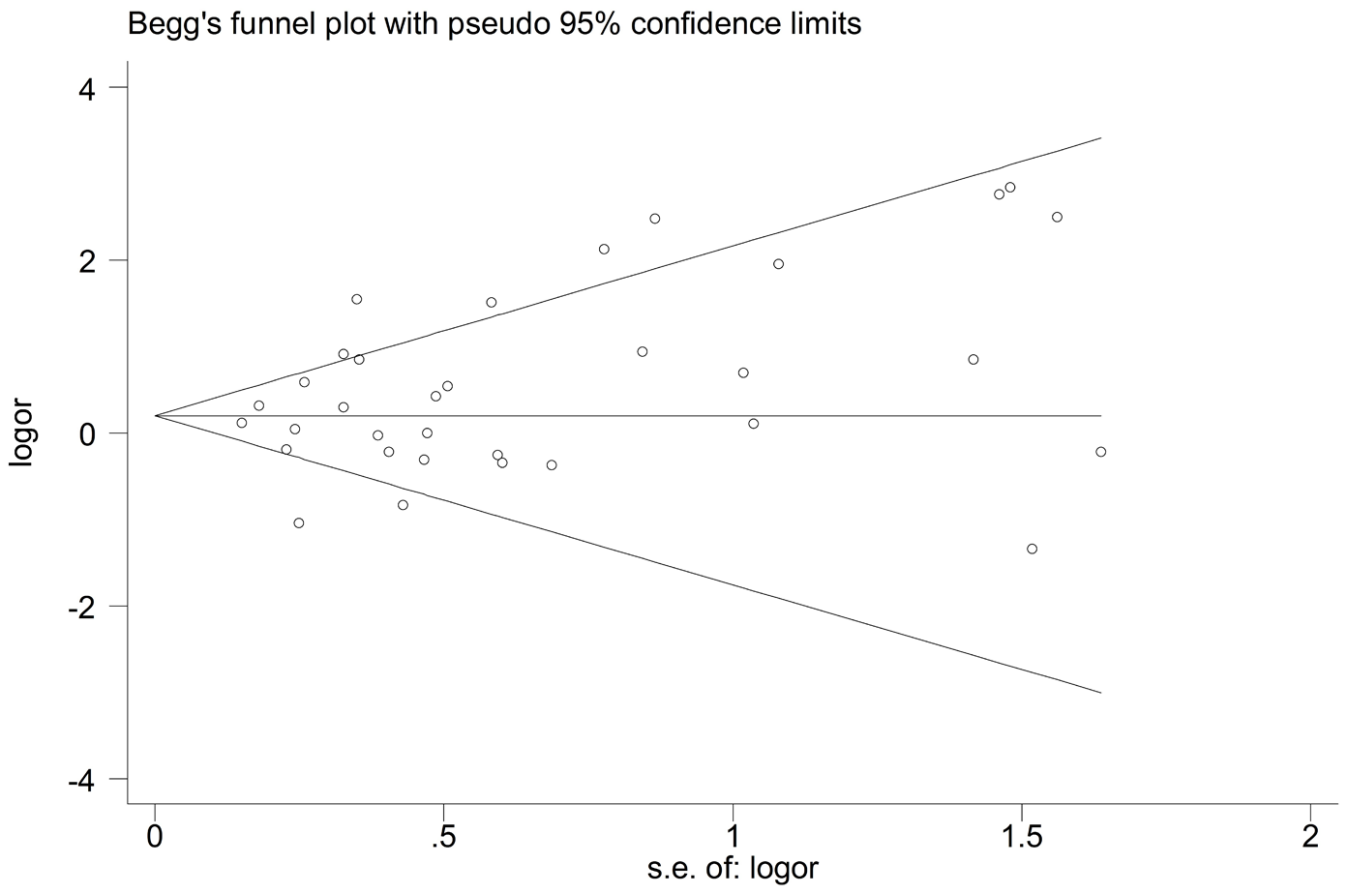
Funnel plot for studies of the association of myocardial infarction and eNOS G894T polymorphism under a homozygous genetic model (TT vs. GG).

Publication bias of the selected articles was assessed by the Begg’s funnel plot and Egger’s test. The shape of the funnel plot did not show obvious publication bias (Figure 5). Similarly, no evidence of publication bias was observed by Egger’s test (*P* = 0.075 for homozygous genetic model; *P* = 0.299 for heterozygous genetic model; *P* = 0.118 for dominant genetic model; *P* = 0.055 for recessive genetic model).

## Discussion

In the current meta-analysis with 8229 cases and 12839 controls, we found that there were significant associations between *eNOS* G894T polymorphism and MI: OR = 1.41 for the homozygous genetic model, OR = 1.12 for the heterozygous genetic model, OR = 1.35 for the recessive genetic model, and OR = 1.18 for the dominant genetic model. Further stratified analysis revealed that the *eNOS* G894T polymorphism was significantly associated with MI in the Asian subgroup (P<0.05), but not in the non-Asian subgroup (P>0.05). The results indicated that ethnicity played important roles in the association of *eNOS* G894T polymorphism with MI risk.

A number of association studies have investigated the association between the *eNOS* G894T polymorphism and the risk of coronary artery disease (CAD), MI, coronary spasms and hypertension [5], [48], [49]. Mechanism study has also suggested the mutation was functional in the production of NO [8]. Human study showed that blood pressure decrease in the *eNOS* 894TT carriers was greater than the other genotypes carriers after the exercise training [50]. Therefore, subjects carrying the *eNOS* 894TT genotype may have low NO in vivo and are more susceptible to endothelial dysfunction, which might increase the risk of MI. The present meta-analysis results of homozygous and recessive genetic models can account for the above hypothesis. Nevertheless, the number of TT genotype is relatively small in Asia populations and the 95%CI line of the pooled OR for Asia populations is longer than that for non-Asia population studies in Figure 1 and 2. So the results of homozygous and recessive genetic models in Asia populations need to be further confirmed in future.

Conflicting results have been reported in investigating the association of the *eNOS* G894T polymorphism with MI. To our knowledge, our meta-analysis represents the first one focusing on the association between *eNOS* G894T polymorphism and the risk of MI. In 2004, Casas et al [51]. performed a meta-analysis to evaluate the association between *eNOS* G894T polymorphism and ischemic heart disease (IHD) including MI and CAD. They found that individuals homozygous for the *eNOS* 894T allele were at moderately increased risk of IHD. In 2012, the meta-analysis results of Zhang indicated that *eNOS* G894T polymorphism was associated with CAD risk among Asia population [5]. However, the above two meta-analysis did not evaluate the association between *eNOS* G894T polymorphism and MI. Our meta-analysis provided a precise result regarding the association of *eNOS* G894T polymorphism with MI risk.

Between-study heterogeneity is common and should be explored in the meta-analysis. In the current study, significant heterogeneity was found in the association of *eNOS* G894T polymorphism with MI risk. Therefore, meta-regression and subgroup analyses were performed to explore the sources of between-study heterogeneity. The results indicated that ethnicity was the source of heterogeneity in the homozygous and recessive genetic models and total sample size was the source of heterogeneity in all genetic models. Sensitivity analysis revealed that the omission of any single study did not have significant impact on the overall meta-analysis estimate. Furthermore, in the meta-analysis, funnel plot did not reflect considerable asymmetry and Egger’s test also indicated no obvious publication bias. All these made the meta-analysis results reliable to some extent.

There are some limitations in this meta-analysis. First, our meta-analysis was based primarily on the unadjusted ORs with 95% CIs and the potential confounding factors were not available. Second, gene-gene and gene-environment interactions may play important roles in the function of *eNOS* G894T polymorphism, but the effect was not addressed in our meta-analysis.

In conclusion, this meta-analysis demonstrated that the *eNOS* G894T polymorphism was associated with increased risk of MI. Further stratification by ethnicity indicated the association between the polymorphism and MI was restricted in the Asians. However, large-scale studies well designed for the gene-gene and gene-environment interactions information are needed to be conducted to elucidate the associations in future.

## Supporting Information

Supplement S1 **PRISMA 2009 Checklist.**
(DOC)

Supplement S2 **PRISMA 2009 Flow Diagram.**
(DOC)

Supplement S3 **Forest plot of myocardial infarction associated with eNOS G894T polymorphism under a heterozygous genetic model (GT vs. GG) stratified by ethnicity.**
(TIF)

Supplement S4 **Forest plot of myocardial infarction associated with eNOS G894T polymorphism under a dominant genetic model (TT/GT vs. GG) stratified by ethnicity.**
(TIF)
