# Supplementary material for: Endothelial Nitric Oxide Synthase Gene G894T Polymorphism and Myocardial Infarction: A Meta-Analysis of 34 Studies Involving 21068 Subjects
Source: PLoS One. 2014 Jan 30;9(1):e87196. doi: 10.1371/journal.pone.0087196 (PMC3907515; doi:10.1371/journal.pone.0087196)
Supplement: Supplement S2 — PRISMA 2009 Flow Diagram. (DOC) [file pone.0087196.s002.doc]

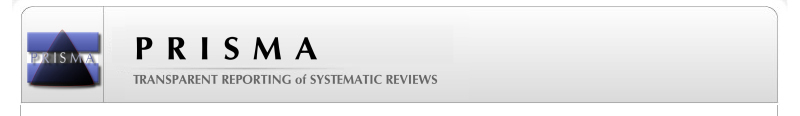
**PRISMA 2009 Flow Diagram**

**Screening**

**Included**

**Eligibility**

**Identification**

Records identified through database searching
(n=417)

Additional records identified through other sources
(n=0)

Records screened
(n=45)

Records excluded:

· Reviews (n=1)

· Duplicated publications (n=4)

· No controls (n=2)

· Insufficient data for calculation of OR (n=4)

Full-text articles assessed for eligibility
(n=34)

Full-text articles excluded for deviation from HWE (n=1)
(n = )

Full-text articles assessed for eligibility
(n=33)

Studies included in quantitative synthesis (meta-analysis)
(n=34)

Data were extracted seperately for each country (n=1)

Records excluded because of obvious irrelevance (n=372)
(n = )
