# Supplementary figures and images for: Endothelial Nitric Oxide Synthase Gene G894T Polymorphism and Myocardial Infarction: A Meta-Analysis of 34 Studies Involving 21068 Subjects
Source: PLoS One. 2014 Jan 30;9(1):e87196. doi: 10.1371/journal.pone.0087196 (PMC3907515; doi:10.1371/journal.pone.0087196)

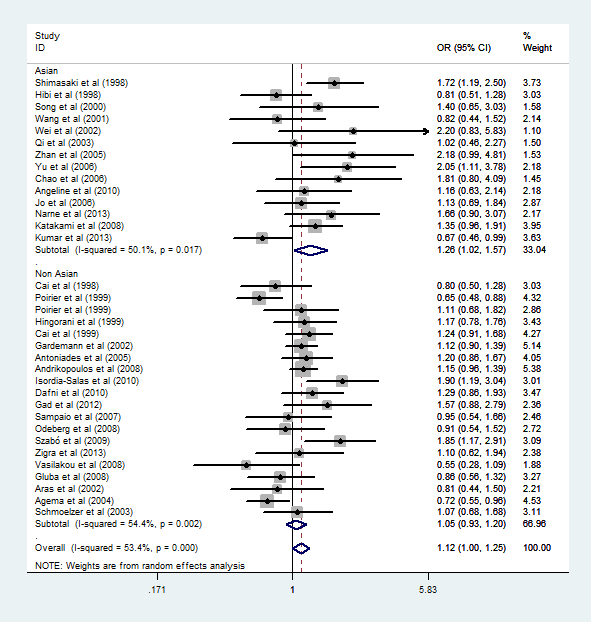

Supplement: Supplement S3 — Forest plot of myocardial infarction associated with eNOS G894T polymorphism under a heterozygous genetic model (GT vs. GG) stratified by ethnicity. (TIF) [file pone.0087196.s003.tif]

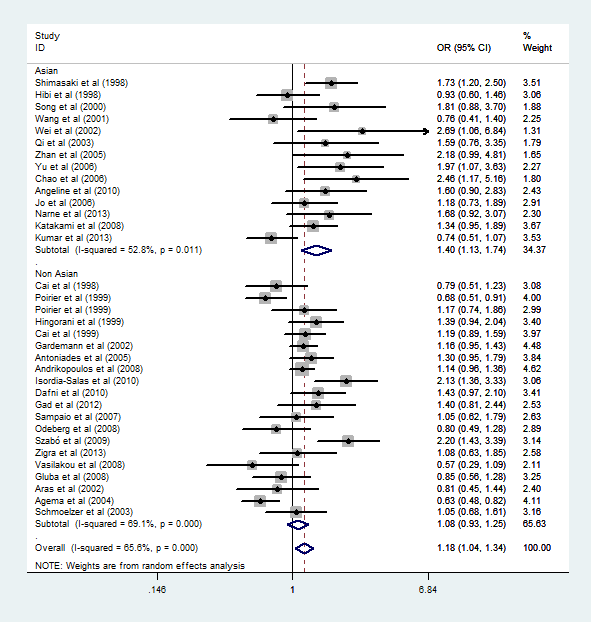

Supplement: Supplement S4 — Forest plot of myocardial infarction associated with eNOS G894T polymorphism under a dominant genetic model (TT/GT vs. GG) stratified by ethnicity. (TIF) [file pone.0087196.s004.tif]
